# Neurochemical properties of BDNF-containing neurons projecting to rostral ventromedial medulla in the ventrolateral periaqueductal gray

**DOI:** 10.3389/fncir.2014.00137

**Published:** 2014-11-20

**Authors:** Jun-Bin Yin, Huang-Hui Wu, Yu-Lin Dong, Ting Zhang, Jian Wang, Yong Zhang, Yan-Yan Wei, Ya-Cheng Lu, Sheng-Xi Wu, Wen Wang, Yun-Qing Li

**Affiliations:** ^1^Department of Anatomy and K.K. Leung Brain Research Centre, Preclinical School of Medicine, Fourth Military Medical UniversityXi'an, China; ^2^Department of Anesthesiology, Fuzhou General Hospital Affiliated to Fujian Medical UniversityFuzhou, China

**Keywords:** periaqueductal gray (PAG), rostral ventromedial medulla (RVM), brain-derived neurotrophic factor (BDNF), projection neurons, neurochemical properties

## Abstract

The periaqueductal gray (PAG) modulates nociception *via* a descending pathway that relays in the rostral ventromedial medulla (RVM) and terminates in the spinal cord. Previous behavioral pharmacology and electrophysiological evidence suggests that brain-derived neurotrophic factor (BDNF) plays an important role in descending pain modulation, likely through the PAG-RVM pathway. However, detailed information is still lacking on the distribution of BDNF, activation of BDNF-containing neurons projecting to RVM in the condition of pain, and neurochemical properties of these neurons within the PAG. Through fluorescent *in situ* hybridization (FISH) and immunofluorescent staining, the homogenous distributions of BDNF mRNA and protein were observed in the four subregions of PAG. Both neurons and astrocytes expressed BDNF, but not microglia. By combining retrograde tracing methods and formalin pain model, there were more BDNF-containing neurons projecting to RVM being activated in the ventrolateral subregion of PAG (vlPAG) than other subregions of PAG. The neurochemical properties of BDNF-containing projection neurons in the vlPAG were investigated. BDNF-containing projection neurons expressed the autoreceptor TrkB in addition to serotonin (5-HT), neurotensin (NT), substance P (SP), calcitonin gene related peptide (CGRP), nitric oxide synthase (NOS), and parvalbumin (PV) but not tyrosine decarboxylase (TH). It is speculated that BDNF released from projection neurons in the vlPAG might participate in the descending pain modulation through enhancing the presynaptic release of other neuroactive substances (NSs) in the RVM.

## Introduction

In the central nervous system, there are multiple neural networks that are involved in pain modulation. The periaqueductal gray (PAG)-rostral ventralmedial medulla (RVM) pathway is located in a pivotal site in the descending pain modulatory system including descending inhibition (DI) and descending facilitation (DF) and also recognized as the site of action for analgesic agents including opioids, cyclooxygenase inhibitors, and cannabinoids (Yaksh et al., 1976; Hohmann et al., 2005; Leith et al., 2007). Now it is generally accepted that the PAG is organized primarily as four longitudinal columns along the rostrocaudal extent: the dorsomedial (dmPAG), dorsolateral (dlPAG), lateral (lPAG), and ventrolateral (vlPAG) subregion of PAG, which is recognized as a heterogenous structure (Paxinos and Watson, 2005). Each column has its own unique neuroanatomical and functional characteristics (Bandler and Shipley, 1994; Behbehani, 1995; Bandler and Keay, 1996). There are various neuroactive substances (NSs) involved in DI or DF in the PAG. Different NSs can produce different effects, and sometimes, the same NS induces both DI and DF (Millan, 2002).

Recent evidence indicates that brain-derived neurotrophic factor (BDNF), a member of the neurotrophin family that is essential for neuronal survival during development, axonal growth, neurotransmission (Birling and Price, 1995; Lewin and Barde, 1996), and contributes to synaptic plasticity in the adult mammalian brain (Thoenen, 1995), could produce both DI and DF in the PAG (Siuciak et al., 1994; Frank et al., 1997; Guo et al., 2006; Zhang et al., 2013). It has been demonstrated that not only do the mRNA and protein of BDNF exist in the PAG (Ceccatelli et al., 1991; Conner et al., 1997), but some BDNF-containing neurons are also observed to project to RVM in the PAG (Guo et al., 2006). The effects of BDNF are mediated through its binding to TrkB and subsequent activation of downstream signaling pathways (Huang and Reichardt, 2003). The BDNF-TrkB signaling pathway has been shown to play a critical role in activity-dependent synaptic plasticity underlying learning and memory through both presynaptic and postsynaptic mechanisms (Schinder and Poo, 2000; Lu, 2003; Carvalho et al., 2008). It is also demonstrated that BDNF could enhance the release of peptides from the primary afferent fibers (Berninger and Poo, 1996; Malcangio et al., 1996). There is evidence indicating that BDNF could facilitate pain through inducing the phosphorylation of N-methyl-D-asparate (NMDA) receptors (Guo et al., 2006), down-regulating the K^+^-Cl^−^ cotransporter (Zhang et al., 2013) and modulating 5-HT (Wei et al., 2010) postsynapticaly in the RVM.

It is important and necessary to investigate the topographic distribution and cell population of BDNF in the PAG for interpreting its related function. The distribution pattern of BDNF-containing projection neurons within the PAG is still unclear. It is necessary to clarify them, because only some subregions of the PAG participate in descending pain modulation (Chen et al., 2008, 2013). In addition, even though infusing BDNF into PAG or RVM can inhibit or facilitate pain (Siuciak et al., 1994; Guo et al., 2006), there is no direct evidence to demonstrate that BDNF is involved in the descending pain modulation through PAG-RVM pathway. Also, there has been no report to investigate the NSs' expression of BDNF-containing projection neurons in the PAG for underling the presynaptic mechanism participating in the descending pain modulation. We therefore observed the neurochemical properties of BDNF-containing projection neurons in the vlPAG.

## Materials and methods

### Animals

Adult male *Sprague Dawley* rats (250–300 g) were used in all experiments. Eighteen rats were divided into 4 groups. Group 1 (3 rats) was used for FISH and double-immunofluorescent histochemical staining. Group 2 (6 rats) was used for simple retrograde tracing investigation and triple-immunohistochemical staining. Group 3 (6 rats) was used for combining retrograde tracing and formalin pain model and triple-immunohistochemical staining. Group 4 (3 rats) was used for injecting normal saline into the hindpaw. Rats were housed in a temperature-controlled environment on a 12 h light/dark cycle with access to food and water *ad libitum*. The experiments were approved by the Institutional Animal Care and Use Committee of the Fourth Military Medical University (Xi'an, China), and the ethical guidelines to investigate experimental pain in conscious animals.

### Fluorescent *in situ* hybridization (FISH) histochemistry

Under deep anesthesia with 2% sodium pentobarbital [100 mg/kg, intraperitoneal (i.p.)], three rats were perfused through the ascending aorta with 200 ml of normal saline containing 0.1% (v/v) diethyl pyrocarbonate (DEPC, DH098-2, Genview, Houston, TX) followed by 500 ml of 2% (w/v) paraformaldehyde containing 15% (v/v) saturated picric acid in 0.1 M phosphate buffer (PB, pH 7.4). The brain was post-fixed for 24 h in the same fixative at 4°C, and transferred to 30% (w/v) sucrose in 0.1 M PB containing 0.1% (v/v) DEPC for 48 h at 4°C. The brain stem was cut into 25 μm thick coronal sections on a freezing microtome (Leica CM1800; Heidelberg, Germany) at −20°C. All processes of FISH were performed following our previous publications (Ge et al., 2014; Kou et al., 2013) and according to the manual (Boster Inc.; Wuhan, China) by using the DNA probe sequences antisense as 5′-GGCGC CACTC CGACC CCGCC CGCCG TGGGG AGCTG-3′ and 5′-AAGTG TAATC CCATG GGTTA CACGA AGGAA GGCTG-3′ for BDNF mRNA. Briefly, free-floating sections were hybridized for 24 h at 50°C with digoxigenin-labeled DNA probe for BDNF in a hybridization buffer. After washes, the hybridized sections were incubated overnight at room temperature (RT) with peroxidase-conjugated antidigoxigenin sheep antibody (11-426-338-910; Roche Diagnostics, Basel, Switzerland) in 0.1 M Tris-HCl (pH 7.5)-buffered 0.9% (w/v) saline containing 1% blocking reagent (TSB). To visualize the signals for BDNF mRNA efficiently, we performed the biotinylated tyramine-glucose oxidase amplification method. Subsequently, the sections were incubated with 10 μg/ml Alexa594-conjugated streptavidin (S-32356; Invitrogen, Eugene, OR) in TSB for 3 h and then incubated for 15 min with DAPI (1:5,000, D1306, Molecular Probes, Eugene, OR, USA) diluted in 0.01 M phosphate-buffered saline (PBS, pH 7.4) and underwent three more wash steps followed by mounting and coverslipping on microscope slides.

Negative controls were treated with hybridization buffer without BDNF DNA probe and the other procedures were unchanged following the previous instructions. No hybridization signals were detected in these sections.

### Intra-RVM stereotaxic microinjections

The injection procedures have been described in our previous study (Chen et al., 2013). In brief, animals were anesthetized with 2% sodium pentobarbital (40 mg/kg, i.p.). A midline opening was made on the skull with a dental drill to insert a glass micropipette (tip diameter 40–60 μm) connected with a microsyringe (1 μl, Hamilton, NV, USA) into the target site. The incisor bar was set at 2.9–3.5 mm below the horizontal plane passing through the interaural line. A volume of 0.06 μl of 4% Fluoro-Gold (FG; Fluorochrome; 80014; Biotium; Hayward, CA, USA) dissolved in normal saline, was pressure-injected into nucleus raphe magnus (NRM), the major part of RVM, according to the following coordinates: 10.5 mm caudal to Bregma, midline and 10.3 mm ventral to the surface of the cranium (Paxinos and Watson, 2005). Each injection was made slowly over 10 min and the injection needle was kept in place for another several minutes. After being kept alive for 5–7 days, the rats were re-anesthetized with 2% sodium pentobarbital (40 mg/kg, i.p.) and injected with 10 μl of 1% colchicines for efficiently detecting the peptide neurotransmitters into the lateral ventricle (0.48 mm caudal to Bregma, 1.6 mm right to midline and 3.6 mm ventral to the surface of the cranium) and survived for another 24 h before perfusion.

### Formalin pain model

In order to test whether the BDNF-containing PAG-RVM pathway was activated by noxious stimuli, formalin pain model was used. After the rats acclimated to a dimly-lit and soundproof testing chamber for about 20 min, they were briefly anesthetized with isoflurane. Then 50 μl of 5% formalin solution (dissolved in normal saline) or 50 μl of normal saline (served as sham control) was subcutaneously (s.c.) injected into the right hindpaw using a microsyringe attached to a 30-G needle after 6–8 d of the FG injection. After s.c. formalin injection, the rats were taken into the observing cage (sound-attenuated, clear Perspex, 25 × 25 × 40 cm) and a video recording was performed for 60 min, as described in previous study (Bai et al., 2012). The pain behaviors were manually recorded with a stop watch by retrieving spontaneous flinches or lickings of the injected hindpaw from the recorded videos. At 2 h after s.c. formalin injection, rats were perfused and used for the later morphological studies.

### Perfusion and tissue preparation

Under deep anesthesia with 2% sodium pentobarbital (100 mg/kg, i.p.), rats were perfused through the ascending aorta with 200 ml of normal saline followed by 500 ml of 2% (w/v) paraformaldehyde and 15% (v/v) saturated picric acid in 0.1 M PB. Then, the brain was removed, immersed in the same fixative for 4 h at 4°C, and transferred to 30% (w/v) sucrose in 0.1 M PB until sink. After being embedded in an inert mounting medium (OCT; Tissue-Tek; Sakura; Torrance, CA, USA), coronal sections of the brain containing PAG or RVM were cut at 25 μm thickness using a freezing microtome and were collected into dishes containing 0.01 M PBS. The sections containing PAG or RVM regions in the first dish were mounted onto gelatin-coated glass slides, air dried and coverslipped with a mixture of 50% (v/v) glycerin and 2.5% (w/v) triethylenediamine (anti-fading agent) in 0.01 M PBS and then observed using a fluorescence microscope (Olympus BX-60; Tokyo, Japan) for investigating the injection or projection sites.

### Double- or triple-labeling immunofluorescence (IF) histochemistry

Following our previous protocol (Li et al., 1996), the sections underwent double- or triple-labeling IF using different antibodies in the combinations and dilutions showing in Table 1. Briefly, free-floating sections containing PAG were blocked for 30 min with 10% normal donkey serum (NDS) in 0.01 M PBS. Then the sections were subjected to the following sequential incubations with: (1) primary antibodies in the antibody dilution medium (0.01 M PBS containing 5% (v/v) NDS (PBS-NDS), 0.3% (v/v) Triton X-100, 0.05% (w/v) NaN_3_ and 0.25% (w/v) λ-carrageenan) overnight at RT and then 72 h at 4°C; (2) a mixture of secondary antibodies in PBS-NDS for 3 h at RT and then 10 h at 4°C; (3) fluorescence isothiocyanate (FITC)-conjugated avidin in PBS containing 0.3% Triton X-100 (PBS-X, pH 7.4), for 2 h at RT.

**Table 1:** **Antibodies used in each group**.

	**Antigen**	**Primary Antibodies**	**Secondary Antibodies**	**Tertiary Antibodies**	**Groups**
Double Staining	BDNF and	Rabbit anti-BDNF (1:200)	Biotin-donkey anti-rabbit (1:500)	FITC-Avidin (1:1,000)	
	NeuN or	Mouse anti-NeuN (1:2,000)	Alexa594-donkey anti-mouse (1:500)		BDNF/NeuN
	GFAP or	Mouse anti-GFAP (1:4,000)	Alexa594-donkey anti-mouse (1:500)		BDNF/GFAP
	OX42 or	Mouse anti-OX42 (1:200)	Alexa594-donkey anti-mouse (1:500)		BDNF/OX42
	FG	Guinea Pig anti-FG (1:300)	Alexa594-goat anti-Guinea pig (1:500)		BDNF/FG
Triple Staining	BDNF and	Rabbit anti-BDNF (1:200)	Biotin-donkey anti-rabbit (1:500)	FITC-Avidin (1:1,000)	
	FG and	Guinea Pig anti-FG (1:300)	Alexa647-donkey anti-Guinea pig (1:500)		
	5-HT or	Goat anti-5-HT (1:500)	Alexa594-donkey anti-goat (1:500)		BDNF/FG/5-HT
	NT or	Rat anti-NT (1:200)	Cy3-donkey anti-rat (1:500)		BDNF/FG/NT
	SP or	Rat anti-SP (1:200)	Cy3-donkey anti-rat (1:500)		BDNF/FG/SP
	CGRP or	Goat anti-CGRP (1:200)	Alexa594-donkey anti-goat (1:500)		BDNF/FG/CGRP
	NOS or	Mouse anti-NOS (1:3,000)	Alexa594-donkey anti-mouse (1:500)		BDNF/FG/NOS
	PV or	Mouse anti-PV (1:1,000)	Alexa594-donkey anti-mouse (1:500)		BDNF/FG/PV
	TH or	Mouse anti-TH (1:5,000)	Alexa594-donkey anti-mouse (1:500)		BDNF/FG/TH
	TrkB or	Goat anti-TrkB (1:100)	Alexa594-donkey anti-goat (1:500)		BDNF/FG/TrkB
	FOS	Mouse anti-Fos (1:500)	Alexa594-donkey anti-mouse (1:500)		BDNF/FG/FOS

Some other sections were used as controls by replacing the primary antibodies with the combinations of normal rabbit, mouse, rat, goat or guinea pig serum according to the species of primary antibodies used, while keeping the other conditions unchanged. (For BDNF, see Supplementary Figure 1).

The antibodies used in the current study were rabbit anti-BDNF antiserum (ab6201; Abcam, Cambridge, MA, USA); mouse anti-NeuN antiserum (MAB377; Millipore, Billerica, MA, USA); mouse anti-GFAP antiserum (MAB3402; Millipore); mouse anti-OX42 antiserum (CBL1512; Millipore); guinea pig anti-FG antiserum (NM-101; PROTOS BIOTECH CORP, New York, NY, USA); goat anti-5-HT antiserum (20079; ImmunoStar, Houston, Texas, USA); rat anti-NT antiserum (NP-103; PROTOS BIOTECH CORP); rat anti-SP antiserum (MAB356; Millipore); goat anti-CGRP antiserum (ab36001; Abcam); mouse anti-NOS antiserum (N-2280; Sigma, St. Louis, MO, USA); mouse anti-PV antiserum (P-3171; Sigma); mouse anti-TH antiserum (T-2928; Sigma); goat anti-TrkB antiserum (sc-20542; Santa Cruz Biotechnology, Santa Cruz, CA, USA); mouse anti-FOS antiserum (ab11959; Abcam); biotin-donkey anti-rabbit IgG (AP182F; Millipore); Alexa594-donkey anti-mouse IgG (A21203; Invitrogen, Carlsbad, CA, USA); Alexa594-goat anti-guinea pig IgG (A-11076; Invitrogen); Alexa594-donkey anti-goat IgG (A-11058; Invitrogen); Cy3-donkey anti-rat IgG (AP189c; Millipore); Alexa647-donkey anti-guinea pig IgG (AP193SA6; Millipore); FITC-Avidin (A-2001; Vector, Burlingame, CA, USA).

### Observation of the fluorescent-positive profiles

After the IF or FISH, the sections were observed and images were captured under confocal laser scanning microscope (FV1000; Olympus) with appropriate filters for FITC (excitation 492 nm, emission 520 nm), Cy3 (excitation 552 nm, emission 565 nm), Alexa594 (excitation 590 nm, emission 618 nm), or Alexa647 (excitation 647 nm, emission 666 nm). For counting the numbers of labeled cells, a careful focus through the thickness of all sections determined that the immunolabeling had penetrated the whole thickness of sections, and only did the neuronal cell bodies, in which the maximum of the nuclei could be observed with obvious light emission, be counted. In addition, the sections were carefully moved across the stage and analyzed from left to right.

### Cell counting

To investigate the percentage of mRNA signals among DAPI positive cells, image analysis software ImageJ (National Institutes of Health, Bethesda, MD) was utilized. This module quantified the number of BDNF mRNA signals per section by thresholding mRNA signals above background levels and by using DAPI staining to differentiate between nuclei. Each subregions of PAG was manually outlined for quantification. To examine the distribution of BDNF protein in the PAG, three frames were randomly chosen (100 × 100 μm) in each subregion of PAG per section following the previous study (Tasset et al., 2012). In each area, the total number of BDNF-immunoreactive (-ir) neurons was counted. Sections were captured digitally with 20 X objective by confocal laser microscope. For analyzing the coexpression of BDNF and other NSs and related proteins, we counted the immunopositive neurons separately in total area of each subregion then calculate the percentages of double- or triple-labeling neurons. Counts were performed by a blinded microscopist. Cell count estimates were presented as mean±SD (mean number of total labeled cells per rat). To evaluate variations in data, a one-way analysis of variance (One-Way ANOVA) was used. The level of statistical significance was set at *P* < 0.05.

## Results

### Distributions of BDNF mRNA and protein in the PAG

**Figure 1:**
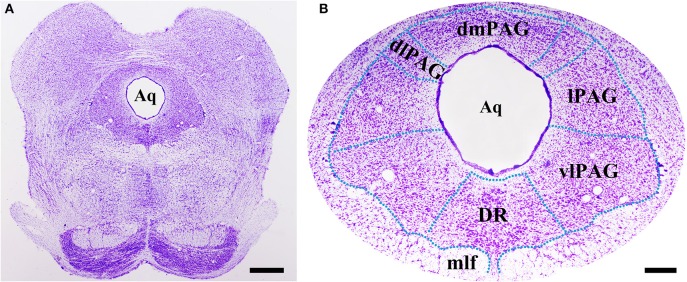
**Nissl staining shows the PAG and its four subregions in the midbrain at Bregma −7.92 mm**. The green dashed lines are manually outlined based on the atlas of the rat brain. Scale bar = 200 μm in **(A)**, 100 μm in **(B)**. Aq, aqueduct; dlPAG, dorsolateral subregion of the PAG; dmPAG, dorsomedial subregion of the PAG; DR, dorsal raphe nucleus; lPAG, lateral subregion of the PAG; mlf, medial longitudinal fasciculus; vlPAG, ventrolateral subregion of the PAG.

**Figure 2:**
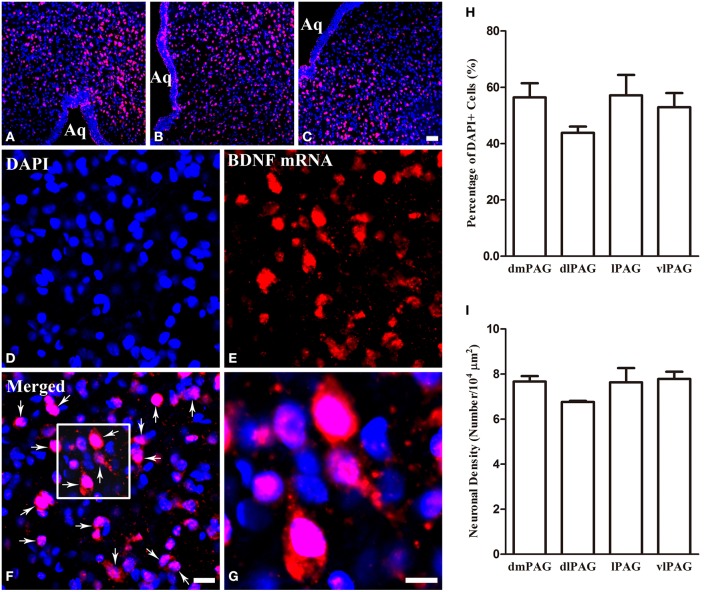
**Distributions of BDNF mRNA and protein in the four subregions of PAG**. **(A–C)** Low magnification FISH photomicrographs show the signals for BDNF mRNA visualizing with Alexa594 (red) and DAPI (blue) in the PAG. **(D–G)** High magnification images also show the signals for BDNF mRNA and DAPI. The framed area in **(F)** is magnified in **(G)**. Arrows indicate cells expressing BDNF mRNA. **(H)** Percentages of BDNF mRNA signals among DAPI^+^ cells in the four subregions of PAG. **(I)** Numbers of BDNF-ir neurons per 100 × 100 μm^2^ in the four subregions of PAG. There was no significant difference between columns in **(H)** and **(I)**. Scale bars = 50 μm in **(C)** (applies **A–C**); 20 μm in **(F)** (applies **D–F**); 10 μm in **(G)**.

Obvious gray matters could be observed around the aqueduct in the Nissl-stained sections, in which there were lots of cell bodies (Figures 1A,B). Combining the Nissl staining and the atlas of the rat brain, the four subregions of PAG were outlined manually in different segments: dmPAG, dlPAG, lPAG, and vlPAG (Paxinos and Watson, 2005). We performed fluorescent *in situ* hybridization (FISH) and immunofluorescence (IF) histochemistry to investigate the topographic distributions of BDNF mRNA and protein in the PAG. BDNF mRNA signals were observed throughout the PAG. DAPI was used to identify the location of BDNF mRNA, and all BDNF mRNA-labeled cells had a marked nucleus (Figures 2A–G). There was no difference on the distribution of BDNF mRNA signals, by calculating the percentage of them among DAPI^+^ cells [Figure 2H: dmPAG, 56.4 ± 8.8%; dlPAG, 43.8 ± 3.9%; lPAG, 57.1 ± 12.6%; vlPAG, 52.9 ± 8.7%; One-Way ANOVA, *F*_(3, 8)_ = 1.373, *P* > 0.05]. The distribution pattern of BDNF protein was similar to that of the mRNA in the PAG (**Figures 5A,D,G**). The BDNF-immunoreactive (ir) neuronal densities among the four subregions were similar [Figure 2 I: dmPAG, 7.7 ± 0.4; dlPAG, 6.8 ± 0.1; lPAG, 7.6 ± 1.1; vlPAG, 7.8 ± 0.6 per 100 × 100 μm^2^; One-Way ANOVA, *F*_(3, 8)_ = 1.573, *P* > 0.05]. These results indicate that BDNF mRNA and protein distribute homogenously in the four subregions of PAG.

### Cell populations of BDNF in the PAG

**Figure 3:**
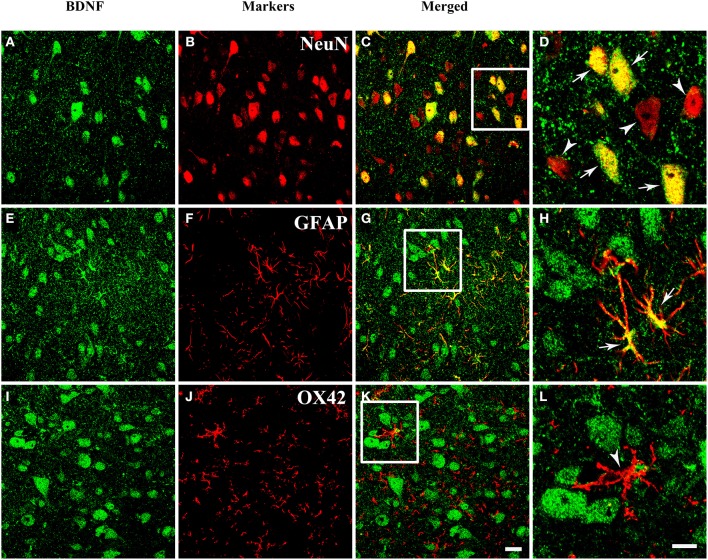
**Fluorescent photomicrographs show both neurons and astrocytes express BDNF, but not microglia**. **(A–D)** Colocalization of BDNF (green) and NeuN (red), NeuN is the marker for neurons. **(E–H)** Colocalization of BDNF (green) and GFAP (red), GFAP is the marker for astrocytes. **(I–L)** Expression of BDNF (green) and OX42 (red), OX42 is the marker for microglia. The framed areas in **(C**,**G**,**K)** are magnified in **(D**,**H**,**L)** respectively. Arrows indicate the colocalization of BDNF/NeuN **(D)** and BDNF/GFAP **(H)**; arrowheads indicate the single expression of NeuN **(D)** or OX42 **(L)**. Scale bars = 20 μm in **(K)** (applies **A–C**, **E–G**, **I–K**); 10 μm in **(H)** (applies **D**,**H**,**L**).

The presence of BDNF mRNA and protein in the PAG inspired us to investigate the cell expression patterns of BDNF. The neuronal and glial expressions of BDNF were explored by markers for different cells. Double labeling between BDNF and NeuN (Neuronal Nuclei), as well as between BDNF and GFAP (Glial Fibrillary Acidic Protein) could be observed in the PAG. But double labeling between BDNF and OX42 was not observed (Figure 3). These results indicate that not only neurons (NeuN positive cells), but also astrocytes (GFAP positive cells) express BDNF in the PAG. Microglia (OX42 positive cells) never shows any BDNF staining. Given the existence of BDNF-containing neurons in the PAG, we wanted to know whether these neurons could project to RVM involved in the descending pain modulation.

### Distribution of neurons projecting to RVM in the PAG

**Figure 4:**
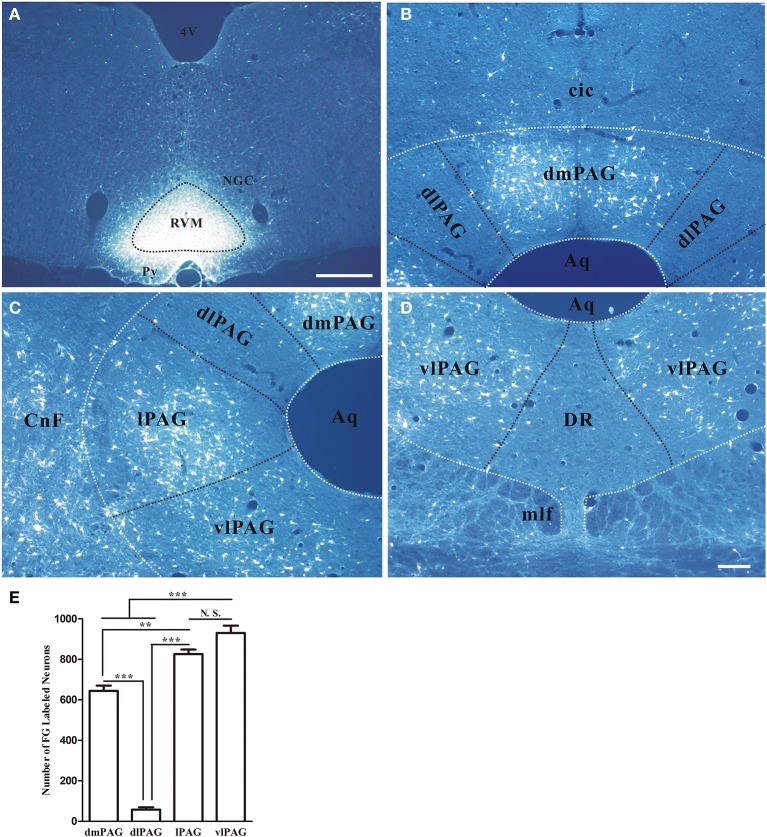
**Distribution of retrogradely labeled neurons in the PAG after FG was injected into the raphe magnus nucleus**. **(A)** Fluorescent photomicrograph shows FG injection site in the raphe magnus nucleus and its adjacent regions. Scale bar = 200 μm. **(B–D)** Fluorescent photomicrographs show the general distributing patterns of FG retrogradely labeled neurons from the RVM in the four subregions of the PAG. FG labeled neurons were observed in the dmPAG **(B)**, lPAG **(C)**, vlPAG **(D)**. In the dlPAG **(B)** and DR **(D)**, few FG labeled neurons were seen. Scale bar = 100 μm. **(E)** Summary of the numbers of FG labeled neurons in four subregions of the PAG. (Mean ± *SD*) N.S. no significant, ^**^ *P* < 0.01, ^***^ *P* < 0.001, One-Way ANOVA, Dunnett's Test. 4V, 4th ventricle; cic, commissure of the inferior colliculus; CnF, cuneiform nucleus; DR, dorsal raphe nucleus; NGC, gigantocellular reticular nucleus; Py, pyramidal tract; RVM, rostroventral medial medulla.

First, the distribution of neurons projecting to RVM in the PAG was examined by using retrograde tracing method. Retrogradely labeled neurons were observed in the PAG after injecting FG into RVM. The representative section showed the injection site was located on the midline and centered in the nucleus raphe magnus (NRM) (Figure 4A). FG labeling was observed in dmPAG, lPAG, and vlPAG. FG-labeled neurons were much less common in dlPAG and dorsal raphe nucleus (DR) (Figures 4B–D). Most FG-labeled neurons were small to medium sized and had fusiform, triangular, oval and/or multipolar shapes. Pressure injection of FG into RVM also gave rise to a large number of retrogradely labeled neurons in the midbrain adjacent to PAG, such as cuneiform nucleus, mesencephalic trigeminal nucleus, ventral tegmental nucleus, and external cortex of the inferior colliculus.

In addition, the numbers of projection neurons in the four subregions of PAG were counted. The numbers of FG-labeled neurons were 644.0 ± 45.0, 58.0 ± 19.1, 825.3 ± 39.1, and 930 ± 62.6, respectively in dmPAG, dlPAG, lPAG, and vlPAG of each rat. There was significant difference among the four subregions [One-Way ANOVA, *F*_(3, 8)_ = 231.8, *P* < 0.0001]. There were more projection neurons in the dmPAG, lPAG, and vlPAG than those in the dlPAG (Figure 4E).

### Distribution of BDNF-containing projection neurons in the PAG

**Figure 5:**
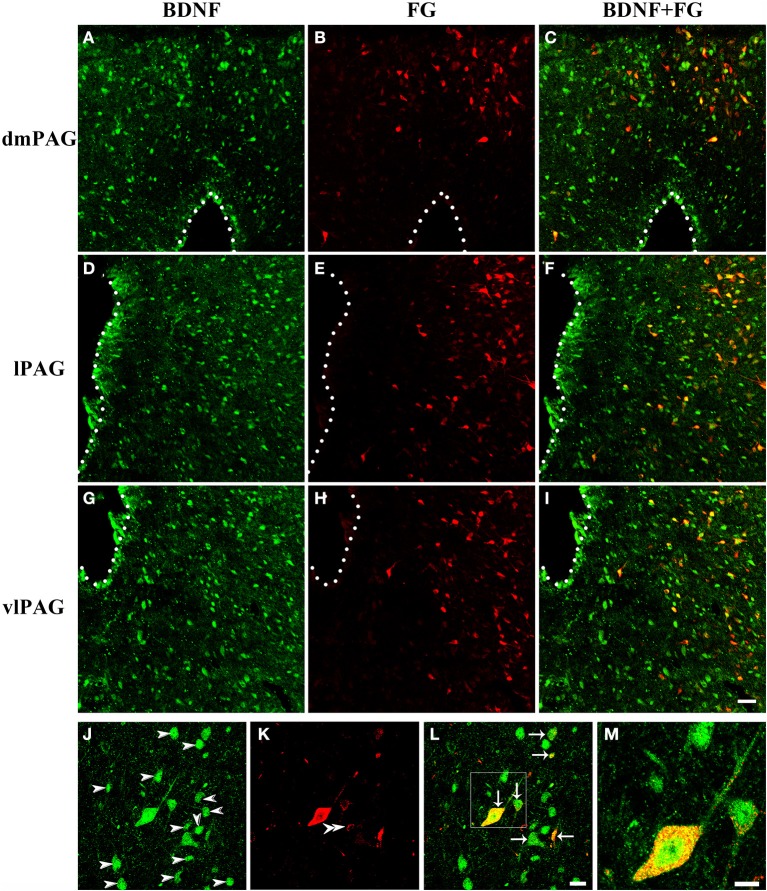
**Fluorescent photomicrographs show the distribution of BDNF-ir neurons and FG labeled neurons in the PAG after FG was injected into the raphe magnus nucleus**. The distributions of BDNF-ir (green) and/or FG labeled (red) neurons are shown in the dmPAG **(A–C)**, lPAG **(D–F)**, and vlPAG **(G–I)**. **(J–M)** High magnification images show the colocalization of BDNF and FG in the PAG. Arrows indicate the merged BDNF/FG neurons, arrowheads indicated the BDNF-ir neurons, and double arrowheads indicate the FG labeled neurons. Scale bars = 50 μm in **(I)** (applies **A–I**); 20 μm in **(L)** (applies **J–L**); 10 μm in **(M)**.

Next, double-labeling IF was used to investigate whether these projection neurons expressed BDNF. BDNF-ir neurons, FG labeled neurons and BDNF/FG-ir neurons were all observed in the PAG (Figure 5). The numbers of BDNF/FG-ir neurons in the dmPAG, dlPAG, lPAG, and vlPAG were 452.0 ± 59.1, 36.3 ± 6.5, 582.3 ± 43.3, and 756.7 ± 34.3, respectively. There was significant difference among the four subregions [One-Way ANOVA, *F*_(3, 8)_ = 170.4, *P* < 0.0001]. The number of BDNF/FG-ir neurons in the vlPAG was more than those in the other subregions (Table 2). Respectively, 18.7 ± 1.4%, 11.4 ± 1.3%, 24.1 ± 0.2%, and 24.0 ± 0.5% of BDNF-ir neurons were BDNF/FG-ir neurons in the dmPAG, dlPAG, lPAG, and vlPAG. There was also significant difference among the four subregions [One-Way ANOVA, *F*_(3, 8)_ = 110.0, *P* < 0.0001]. The proportion in the vlPAG was higher than those in the dmPAG or dlPAG (Table 2). The proportions of BDNF/FG-ir neurons to FG labeled neurons were 70.6 ± 3.0%, 65.8 ± 8.4%, 70.6 ± 5.4%, and 81.5 ± 0.9%. Similar to above results, there was significant difference among the four subregions [One-Way ANOVA, *F*_(3, 8)_ = 4.8, *P* < 0.05]. And the proportion in the vlPAG was higher than that in the dlPAG (Table 2). According to these results, it is supposed that there is factually a PAG-RVM BDNF-containing pathway and BDNF-containing projection neurons are likely to be distributed over most but not all of the PAG.

**Table 2:** **Numbers of neurons labeled with BDNF and/or FG in four subregions of the PAG**.

	**dmPAG**	**dlPAG**	**lPAG**	**vlPAG**
Neurons singly labeled with BDNF only	1960.7±83.2	282.3±25.5	1848.7±98.7	2392.0±86.7
Neurons singly labeled with FG only	191.0±49.8	18.7±4.2	242.3±48.2	172.3±18.0
Neurons dually labeled with BDNF and FG	452.0±59.1 ^a1^	36.3±6.5^a2^	582.3±43.8^a3^	756.7±34.3
Neurons labeled with BDNF	2412.7±141.7	318.7±30.7	2411.0±168.1	3148.7±117.1
Neurons labeled with FG	643.0±108.3	55.0±4.4	824.7±26.7	929.0±52
Ration of dually labeled neurons in BDNF neurons	18.7±1.4%^b1^	11.4±1.3%^b2^	24.1±0.2%^b3^	24.0±0.5%
Ration of dually labeled neurons in FG neurons	70.6±3.0%^c1^	65.8±8.4%^c2^	70.6±5.4%^c3^	81.5±0.9%

*Data are shown as Mean ± SD, N = 3*.

a1,a2 *P < 0.0001*,

a3 *P < 0.001*,

b1,b2 *P < 0.0001*,

b3 *P > 0.05*,

c1,c3 *P > 0.05*,

c2 *P < 0.05*,

*One-Way ANOVA, Dunnett's Test*.

### Activation of BDNF-containing projection neurons in the formalin model

**Figure 6:**
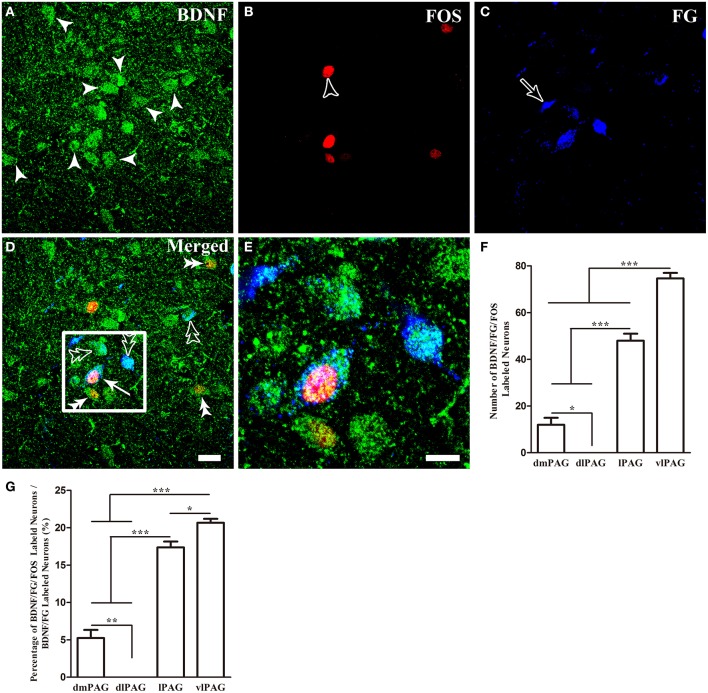
**Activation of BDNF-containing projection neurons in the PAG after formalin injection**. BDNF-containing (green) projection neurons (blue) could express FOS (red). Arrows indicate BDNF/FG/FOS merged neurons **(D)**; filled arrowheads indicate neurons singlely expressing BDNF **(A)**; unfilled arrowheads indicate neurons singlely expressing FOS **(B)**; unfilled arrows indicate neurons singlely labeled by FG **(C)**; double filled arrowheads indicate colocalization of BDNF and FOS **(D)**; double unfilled arrowheads indicate BDNF-ir and FG labeled neurons **(D)**. The framed areas in **(D)** are magnified in **(E)**. Counting the numbers of activated BDNF-containing projection neurons (BDNF/FG/FOS-ir neurons) **(F)** and the percentages of these neurons among BDNF-containing projection neurons **(G)** in the four subregions of PAG. ^*^ *P* < 0.05, ^**^ *P* < 0.01, ^***^ *P* < 0.001, One-Way ANOVA, Dunnett's Test. Scale bars = 20 μm in **(D)** (applies **A–D**); 10 μm in **(E)**.

Finally, we sought to identify whether these BDNF-containing projection neurons in the PAG were involved in pain modulation. The activation of these neurons was indicated by FOS expression after formalin was injected into the hindpaw. Two hours after formalin injection, FOS labeling neurons were characterized with densely stained nuclei, which were round or oval with unstained cytoplasm. In many regions of the midbrain related to pain modulation, FOS could also be found, such as DR, median raphe nucleus (MnR), paramedian raphe nucleus (PMR), interpeduncular nucleus (IPN), superior and inferior colliculus (Supplementary Figure 2). In the PAG, FOS was also observed, especially in the caudal vlPAG. The numbers of BDNF/FG/FOS labeled neurons were 12 ± 5.2, 0, 48 ± 5.2, 74.7 ± 4.1 and the portions of these neurons to BDNF/FG labeled neurons were 5.3 ± 1.9%, 0, 17.4 ± 1.3%, 20.6 ± 0.9% in the dmPAG, dlPAG, lPAG, vlPAG, respectively. There were significant differences among the four subregions [One-Way ANOVA, *F*_(3, 8)_ = 198.4, *P* < 0.0001; *F*_(3, 8)_ = 181.8, *P* < 0.0001] (Figure 6). In addition, the number of BDNF/FG/FOS labeled neurons and the activated percentage in the vlPAG were more than those in the other subregions. In the sections from control rats, the distribution of FOS labeled neurons was sparse and only scattered FOS was found in the PAG. And less than 1% of BDNF-ir neurons were labeled with FOS in the PAG. It is suggested that BDNF-containing projection neurons in the PAG play a role in the formalin-induced hindpaw pain and vlPAG might play a more important part than the other subregions. So our attentions were focused on the BDNF-containing projection neurons in the vlPAG.

### Neurochemical properties of BDNF-containing projection neurons in the vlPAG

**Figure 7:**
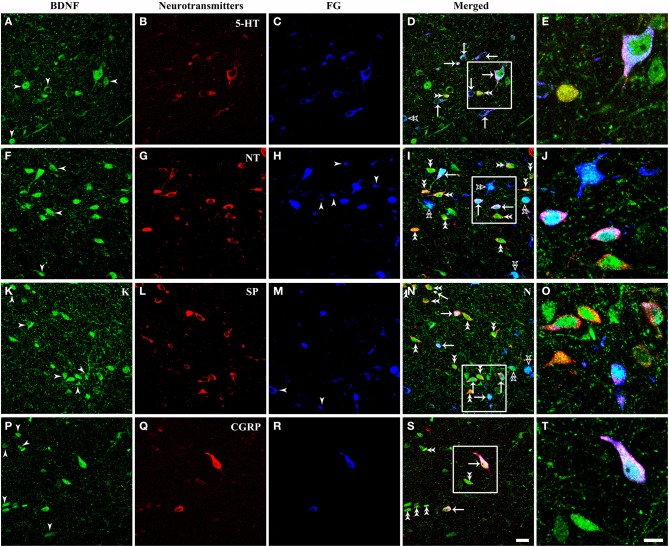
**Fluorescent photomicrographs show the expression of neurotransmitters (5-HT, NT, SP, CGRP) in BDNF-containing projection neurons in the vlPAG after FG was injected into the raphe magnus nucleus**. BDNF-containing (green) projection neurons (blue) could express 5-HT **(A–E)**, NT **(F–J)**, SP **(K–O)**, CGRP **(P–T)** (red). Arrows indicate the merged BDNF/FG/neurotransmitters, BDNF-ir projecting neurons expressing 5-HT **(D)**, NT **(I)**, SP **(N)**, CGRP **(S)**; filled arrowheads indicate singly BDNF-ir **(A,F,K,P)** or FG labeled neurons **(H,M)**; double filled arrowheads indicate BDNF and 5-HT **(D)**, NT **(I)**, SP **(N)**, CGRP **(S)** merged neurons; double unfilled arrowheads indicate BDNF and FG merged neurons **(D,I,N)**. The framed areas in **(D,I,N,S)** are magnified in **(E,J,O,T)** respectively. Scale bars = 20 μm in **(S)** (applies **A–D**,**F–I**, **K–N**, **P–S**); 10 μm in **(T)** (applies **E,J,O,T**).

**Figure 8:**
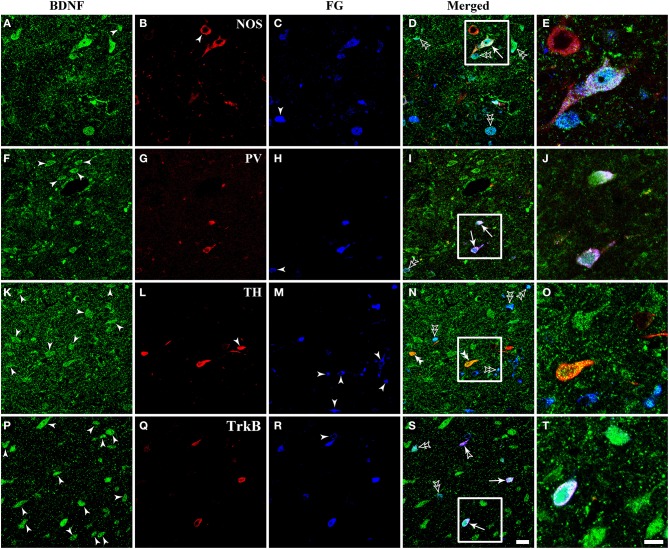
**Fluorescent photomicrographs show the expression of NOS, PV, TH, and TrkB in BDNF-containing projection neurons in the vlPAG after FG was injected into the raphe magnus nucleus**. BDNF-containing (green) projection neurons (blue) could express NOS, PV, and TrkB, but not TH (red). Arrows indicate the triply labeled neurons, BDNF-ir projecting neurons expressing NOS **(D)**, PV **(I)**, and TrkB **(S)**; filled arrowheads indicate singly BDNF- **(A,F,K,P)** or NOS- **(B)** or TH-ir **(L)** or FG **(C,H,M,R)** labeled neurons; double filled arrowheads indicate BDNF and TH **(N)** merged neurons; double unfilled arrowheads indicate BDNF and FG **(D,I,N,S)** merged neurons; filled and unfilled arrowheads indicate FG and TrkB merged neurons **(S)**. The framed areas in **(D,I,N,S)** are magnified in **(E)**, **(J,O,T)** respectively. Scale bars = 20 μm in **(S)** (applies **A–D, F–I, K–N, P–S**); 10 μm in **(T)** (applies **E,J,O,T**).

It is important for comprehending the roles of BDNF in the descending pain modulation to investigate chemical features of BDNF-containing projection neurons in the vlPAG. Triple-labeling IF was used to examine the colocalization of BDNF, FG and serotonin (5-HT), neurotensin (NT), substance P (SP), calcitonin gene related peptide (CGRP), nitric oxide synthase (NOS), parvalbumin (PV), or tyrosine decarboxylase (TH) that characterized BDNF-containing projection neurons in the vlPAG (Figures 7, 8). 5-HT-ir neurons were observed mainly in the DR and vlPAG, and diffused distribution in the dmPAG and lPAG. SP-ir neurons could be observed in the lPAG, CnF, vlPAG and DR, especially in the DR and lPAG. NT-ir neurons and fibers were observed around the PAG, of which DR, lPAG, and vlPAG had the most numbers of NT-ir neurons. Numerous CGRP-ir neurons that were fusiform, spheroidal, and triangular in shape were observed, mostly in the caudal vlPAG and DR in the rostral PAG. And BDNF-containing projection neurons expressing 5-HT, NT, SP and CGRP could also be observed (Figure 7).

There were numerous neurons expressing NOS in the dlPAG, vlPAG, and caudal DR. PV-expressing neurons were observed in all four subregions of the PAG and DR. The TH-ir neurons mainly distributed in the DR, and small numbers extended into the vlPAG of the rostral portion. BDNF-containing projection neurons expressing NOS and PV could be observed, while triplely labeled BDNF/FG/TH neurons were not found (Figure 8).

The numbers and percentages of these triple- and double-labeling neurons were shown in Table 3. The numbers of triple-labeling neurons were 280.0 ± 37.7, 366.7 ± 45.4, 309.3 ± 12.2, 304 ± 27.7, 106.7 ± 9.2, and 212.7 ± 12.7 for 5-HT, NT, SP, CGRP, NOS, and PV. Among BDNF/5-HT-ir, BDNF/NT-ir, BDNF/SP-ir, BDNF/CGRP-ir, BDNF/NOS-ir, BDNF/PV-ir neurons, there were 70.4 ± 6.2%, 34.2 ± 5.7%, 39.6 ± 5.6%, 33.7 ± 3.6%, 30.9 ± 3.9%, 69.1 ± 2.4% neurons that projected to RVM, respectively. In the vlPAG, 38.8 ± 6.8%, 47.0 ± 6.5%, 44.8 ± 2.9%, 42.7 ± 3.5%, 14.7 ± 0.9%, 28.5 ± 1.6% of BDNF-containing projection neurons expressed 5-HT, NT, SP, CGRP, NOS, PV, respectively. BDNF could be expressed in 84.0 ± 4.9%, 88.2 ± 2.9%, 87.3 ± 2.6%, 85.5 ± 4.8%, 69.3 ± 8.9%, 70.9 ± 6.3% of 5-HTergic, NTergic, SPergic, CGRPergic, NOS-containing, PV-containing projection neurons in the vlPAG.

**Table 3:** **Numbers of neurons labeled with BDNF/5-HT, NT, SP, CGRP, NOS, PV, TH, TrkB/FG in the vlPAG**.

	**5-HT**	**NT**	**SP**	**CGRP**	**NOS**	**PV**	**TH**	**TrkB**
Neurons triply labeled with BDNF/5-HT, NT, SP, CGRP, NOS, PV, TH, TrkB/FG	280.0±37.7	366.7±45.4	309.3±12.2	304±27.7	106.7±9.2	212.7±12.7	0	443.3±45.0
Neurons dually labeled with BDNF/5-HT, NT, SP, CGRP, NOS, PV, TH, TrkB	400.0±62.4	1076.7±75.9	781.3±20.1	904±18.3	346.7±24.4	308.0±22.0	130.7±16.2	625.3±16.2
Neurons dually labeled with BDNF/FG	725.0±31.2	781.7±32.1	690.7±24.4	712±6.9	725.3±18.5	748.0±38.1	737.3±64.7	718.7±8.1
Neurons dually labeled with 5-HT, NT, SP, CGRP, NOS, PV, TH, TrkB/FG	335.0±56.8	415.0±39.1	354.7±24.4	356±30.2	154.7±9.2	300.7±12.7	9.3±16.2	546.0±50.5
Ration of triply labeled neurons in neurons dually labeled with BDNF/5-HT, NT, SP, CGRP, NOS, PV, TH, TrkB (%)	70.4±6.2	34.2±5.7	39.6±5.6	33.7±3.6	30.9±3.9	69.1±2.4		70.8±6.3
Ration of triply labeled neurons in neurons dually labeled with BDNF/FG (%)	38.8±6.8	47.0±6.5	44.8±2.9	42.7±3.5	14.7±0.9	28.5±1.6		61.7±5.9
Ration of triply labeled neurons in neurons dually labeled with 5-HT, NT, SP, CGRP, NOS, PV, TH, TrkB/FG (%)	84.0±4.9	88.2±2.9	87.3±2.6	85.5±4.8	69.3±8.9	70.9±6.3		81.2±1.3

*Data are shown as Mean ± SD, N = 3*.

### Expression of TrkB in BDNF-containing projection neurons

Respecting BDNF-containing projection neurons expressed 5-HT, NT, SP, CGRP, NOS, or PV. Besides, BDNF has the ability to enhance the release of neurotransmitters. We next examined whether BDNF-containing projection neurons expressed BDNF receptors, TrkB. Using the same approach, TrkB-containing neurons were observed in all of the four subregions of PAG and DR. In the vlPAG, there were 443.3 ± 45.0 BDNF/TrkB/FG-ir neurons, 70.8 ± 6.3% of BDNF/TrkB-ir neurons projected to the RVM, 61.7 ± 5.9% of BDNF-containing projection neurons expressed TrkB, and 81.2 ± 1.3% of TrkB-containing projection neurons expressed BDNF. These results indicate that BDNF and its receptor TrkB coexist in the same PAG-RVM projection neurons.

## Discussion

The present data enhance our understanding of the PAG-RVM pathway underlying the descending pain modulation, including descending inhibition and facilitation. Specifically, the data demonstrate that (i) in the PAG, BDNF homogeneously distributes in the four subregions and is mainly expressed in neurons and astrocytes; (ii) a subset of PAG neurons expressing BDNF are involved in the formalin pain model especially in the vlPAG, which also projecte to RVM; (iii) in the vlPAG, BDNF-containing neurons projecting to RVM express 5-HT, NT, SP, CGRP, NOS, or PV; (iv) BDNF-containing projection neurons also express its own receptors, TrkB. It is speculated that BDNF released from projection neurons in the vlPAG participate in the descending pain modulation likely through regulating the presynaptic release of NSs in the RVM.

### Distributions and cell populations of BDNF in the PAG

Immunohistochemistry and *in situ* hybridization in bright-field method have been used to show that neurons in the PAG and associated regions express BDNF (Ceccatelli et al., 1991; Conner et al., 1997). However, these studies have not specifically focused on the PAG and have, therefore, not considered the precise distribution of BDNF in the four subregions of PAG. We used both FISH and IF to detect the mRNA and protein in the PAG and compared the distributions of BDNF in the four subregions. About half of the DAPI^+^ cells in the PAG expressed mRNA of BDNF, and there were 7.5 ± 0.7 BDNF-ir neurons per 100 × 100 μm^2^. There were no significant differences on the distributions of BDNF mRNA and protein among the four subregions of PAG. These homogeneous distributions reflect the multifunctionality of BDNF in the mammalian brain, including neuronal survival during development, axonal growth, neurotransmission, and modulation of synaptic plasticity (Birling and Price, 1995; Thoenen, 1995; Lewin and Barde, 1996). Our report now clarifies which cells express BDNF in the PAG.

Neurons, astrocytes, and microglia all express BDNF in other different regions of brain (Martinowich et al., 2007; Boyadjieva and Sarkar, 2013; Degos et al., 2013; Gomes et al., 2013; Quesseveur et al., 2013), but microglia do not express BDNF in the PAG. What is the reason of expressing cellular differences, especially between astrocytes and microglia in different regions? Heterogeneity of BDNF expression might confer a regional specificity on cellular expressing of BDNF for different functions. For example, BDNF released from astrocytes might locally modulate local circuits that participate in the descending targets of the PAG. The neuronal expression of BDNF illustrated the probability that BDNF-containing neurons could project to the RVM and participate in descending pain modulation.

### Distribution and activation of BDNF-containing projection neurons in the PAG

In the present study as well as in previous reports using different retrograde tracers including horseradish peroxidase (HRP) and colloidal gold-labeled wheat germ agglution conjugated to HRP (WGA-HRP) (Gallager and Pert, 1978; Carlton et al., 1983), neurons retrogradely labeled from the RVM were distributed throughout the dmPAG, lPAG, vlPAG, and were relatively less common in the dlPAG and DR. In the present study, projection neurons in the PAG expressed BDNF, consistent with a previous study using cholera toxin subunit B (CTB) as retrograde tracer (Guo et al., 2006). However, the coexpression of BDNF and CTB was only investigated in the vlPAG and lPAG. In addition to this, the numbers of BDNF/FG-ir neurons in the dlPAG and dmPAG were counted in our study. Although FG has the similar efficiency of labeling to CTB used for retrograde tracing (Spike et al., 2003), we detected more BDNF-ir neurons due to different antibody against BDNF used in our work. Therefore higher percentages of BDNF-ir projection neurons among FG labeled neurons were observed, 81.5 ± 0.9% and 70.6 ± 5.4% vs. 63 ± 4% and 41 ± 6%, in the vlPAG and lPAG respectively.

Even though it has been reported that exogenously applied BDNF into the PAG produces analgesia (Siuciak et al., 1994; Frank et al., 1997) or inflammatory pain induces increase in BDNF expression in PAG neurons (Guo et al., 2006), there is no direct evidence for activation of BDNF-containing projection neurons in the PAG. Furthermore, which subregions of PAG BDNF-containing projection neurons are involved in descending pain modulation? In our investigation, the number of activated BDNF-containing projection neurons and the percentage of them among BDNF-containing projection neurons in the vlPAG were higher than those in the other subregions in the formalin pain model. In the previous studies (Morgan, 1991; Guo et al., 2006), the vlPAG has been shown to be pivotal in modulation of morphine-induced analgesia and descending facilitation in the inflammatory pain. The vlPAG is thought to be the most important part for descending pain modulation and great parts of modulatory effects in other subregions are achieved by changing the activity of neurons in the vlPAG, although analgesic effect can be fulfilled through stimulating the four subregions of PAG (Bandler and Depaulis, 1991; Millan, 2002). It should also be noticed that BDNF-containing projection neurons were activated in dmPAG and lPAG. Those functional BDNF-containing projection neurons might be related to fear and anxiety, and vocalization for pain rather than pain modulation.

### Neurochemical properties of BDNF-containing projection neurons in the vlPAG

There are lots of NSs involved in the PAG-RVM descending pathway, which is thought to be one reason for complexity of descending pain modulatory system (Millan, 2002; Fu et al., 2010; Bowman et al., 2013). Glutamate and GABA are the most widely distributed neurotransmitters in the central nervous system. There are numerous neurons containing WGA-HRP and glutamate in the PAG after injecting the WGA-HRP into the NRM (Beitz, 1990). Although the numbers and proportions of double labeled neurons were not reported in that study, approximately 40% of myelinated axons in the NRM contained glutamate-like immunoreactivity and glutamate-ir was also observed in unmyelinated axons. GABAergic neurons are small in size, make up about 15–20% of all PAG neurons (Barbaresi and Manfrini, 1988; Reichling and Basbaum, 1990). However, only 1.5% of all retrogradely labeled neurons from the NRM in the PAG are also GABA-ir (Reichling and Basbaum, 1990). It is necessary that the coexpression of glutamate or GABA/BDNF/FG needed to have been investigated. However, using antibodies we failed to detect glutamate, GABA, or markers for them in the PAG. In the future, we hope to observe the coexpression of BDNF/glutamate or GABA through ISH or using transgenic animals. There are also other NSs in the PAG, including enkephalin, SP, somatostatin, galanin, vasoactive intestinal polypeptide, neuropeptide Y, CGRP, 5-HT, NT, NO, dopamine, cholecystokinin, and acetycholine, etc. (Smith et al., 1994; Millan, 2002). However, there is not one review summarizing the neurochemical properties of PAG and those proportions. For these reasons, we chose those that had been investigated more involved in the pain modulation. Moreover, the percentages of BDNF/FG/NSs-ir neurons among the BDNF/FG-ir neurons were not low in our present investigation. For other NSs that were not investigated in our sudy, some were concerned less, such as vasoactive intestinal polypeptide, some were not involved in the PAG-RVM pathway, such as cholecystokinin (Beitz et al., 1983), some were thought involved in other functions of PAG, such as acetycholine (Deolindo et al., 2011). Of course, the NSs investigated in our study are a part of all NSs involved in the PAG-RVM pathway. More studies about other NSs are indeed to be carried out in the future.

The NSs-ir neurons had a similar distribution as previous studies (Beitz, 1982b,a; Smith et al., 1994; Chen et al., 2003; Abrams et al., 2004; Fu et al., 2010). Some of these neurons can inhibit and/or facilitate pain, leading to analgesia and/or hyperalgesia. Increasing 5-HT synthesis facilitates analgesia, whereas decreasing 5-HT synthesis or blocking 5-HT-receptor-mediated transmission attenuates analgesia (Basbaum and Fields, 1984; Stamford, 1995). Neurokinin-1 receptor (NK-1R)-ir neurons in RVM are modulated by SP-ir efferent fibers from the PAG (Zeng et al., 1991; Chen et al., 2013), and is essential for the full expression of descending pain modulation (De Felipe et al., 1998; Bester et al., 2001). Dose response analyses revealed that NT produced intensive response at lower dose in addition to analgesia at higher doses following intra-RVM administration (Fang et al., 1987; Urban and Smith, 1993). The hindpaw withdrawal latency to nociceptive stimulation increases significantly after intra-RVM administration of CGRP, which can be antagonized by its receptor antagonist CGRP8-37 (Huang et al., 2000).

Some NSs are difficult to examine directly. An alternate approach is to examine key enzymes that synthesize or otherwise mark the presence of those NSs. NO is produced from L-argine by NOS, and distributed in the PAG (Onstott et al., 1993; Fu et al., 2010). NO in the vlPAG plays an important role in visceral pain modulation and defensive reactions (Hamalainen and Lovick, 1997; Rodella et al., 1998; Emmanouil et al., 2008). PV, a calcium-binding protein, is involved in the control of intracellular calcium homeostasis in inhibitory neurons (Nitsch et al., 1990). In the PAG, inhibitory neurons make the fundament for opioidergic descending analgesia (Park et al., 2010). TH is the rate-limiting enzyme in the biosynthesis of catecholamines, which is a marker of dopamine (Nagatsu et al., 1964). PAG dopamine has a direct antinociceptive effect in addition to modulating the analgesic effect of morphine (Meyer et al., 2009). Surprisingly, coexpression of BDNF/TH/FG was not observed in the vlPAG. The possible reason was that few TH-ir neurons could project from the vlPAG to the RVM, which was coincidence with the previous study (Suckow et al., 2013).

### Functional consideration of BDNF-containing projection neurons expressing TrkB in the vlPAG

Release of NSs, is modulated within milliseconds to seconds through action on presynaptic receptors (Merighi, 2002; Carvalho et al., 2008). Abundant mRNA and protein of TrkB are observed in the central nervous sysem (Yan et al., 1997; King et al., 1999). Full-length TrkB, BDNF, and SP or CGRP-ir central terminals of primary afferent fibers make synapses with dendrites (Salio et al., 2005). Employing patch-clamp recordings and calcium imaging, it has been demonstrated that BDNF could enhance the release of SP or CGRP by acting on the presynaptic TrkB receptors. Effects can also be blocked by Trk antagonist K252a or anti-TrkB antibody (Merighi et al., 2008). Although there were technical limitations to investigate coexpression of BDNF/TrkB/NSs/FG in the vlPAG, we infer the existence of at least some coexpression for 5-HT, NT, SP and CGRP based on the high percentages of triple-labeling neurons among BDNF/FG-ir neurons. It is presumed that there is the same mechanism for BDNF modulating the presynaptic NSs' release by binding presynaptic TrkB in the PAG-RVM pathway.

Based on the distribution of TrkB in the PAG and the fact that not only neurons but also astrocytes expressed BDNF and directly infusing BDNF into the PAG produces analgesia (Siuciak et al., 1994; Frank et al., 1997), it is presumed that there is a postsynaptic effect for BDNF. BDNF directly activates TrkB receptor localized on neighboring or just the same neurons within the vlPAG except for being transported anterogradely to RVM, as BDNF released from microglia acts on TrkB localized on neurons in the spinal cord (Coull et al., 2005). BDNF can increase the synthesis, transport, or release of NSs in the PAG, through postsynaptic TrkB binding (Croll et al., 1994; Nawa et al., 1994).

In summary, BDNF is expressed mainly in neurons and astrocytes within the PAG. There are amounts of BDNF-containing projection neurons in the four subregions of PAG, however, these neurons in the vlPAG are more activated in the formalin pain model. Furthermore, the vlPAG BDNF-containing neurons projecting to RVM express 5-HT, NT, SP, CGRP, NOS, PV, and TrkB. These findings suggest that BDNF released from the projection neurons in the vlPAG regulates the release of NSs via the presynaptic TrkB. These NSs work together with BDNF participating in the PAG-RVM descending pain modulation.

### Conflict of interest statement

The authors declare that the research was conducted in the absence of any commercial or financial relationships that could be construed as a potential conflict of interest.
