# Supplementary material for: Neurochemical properties of BDNF-containing neurons projecting to rostral ventromedial medulla in the ventrolateral periaqueductal gray
Source: Front Neural Circuits. 2014 Nov 20;8:137. doi: 10.3389/fncir.2014.00137 (PMC4238372; doi:10.3389/fncir.2014.00137)
Supplement: Supplementary file 1 [file DataSheet1.DOCX]

**Supplementary Figures**

**Supplementary Figure 1**


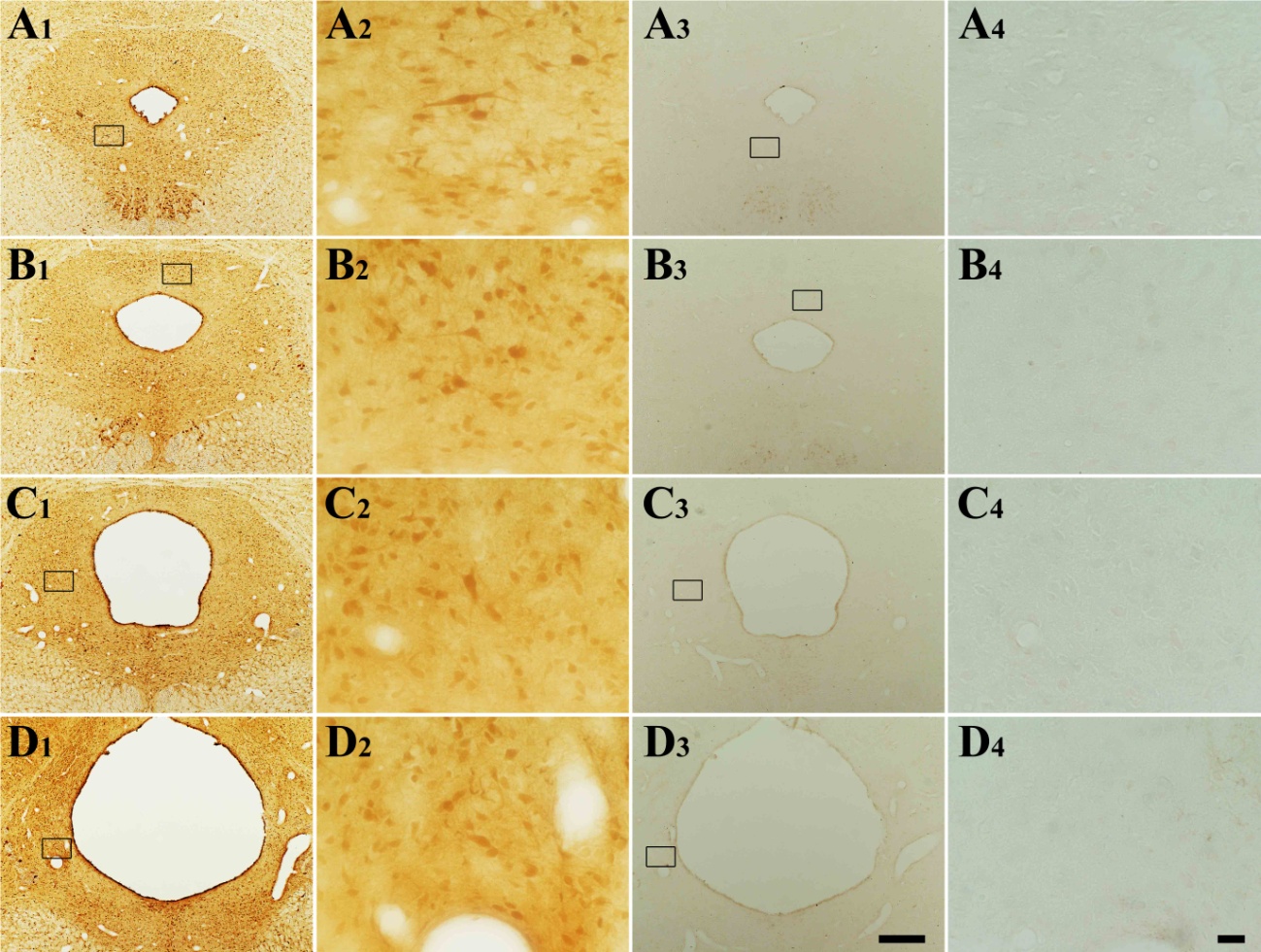


**Photomicrographs of representative PAG sections show the specificity of the BDNF antibody using DAB reaction.** The PAG sections of different midbrain segments are incubated with rabbit anti-BDNF antibody (A_1_, B_1_, C_1_, D_1_). The framed areas in (A_1_), (B_1_), (C_1_), (D_1_) are magnified in (A_2_), (B_2_), (C_2_), (D_2_) respectively. The adjacent sections are incubated with rabbit serum without BDNF antibody (A_3_, B_3_, C_3_, D_3_). The framed areas in (A_3_), (B_3_), (C_3_), (D_3_) are magnified in (A_4_), (B_4_), (C_4_), (D_4_) respectively. Scale bars = 200 μm in D_3_ (applies A_1_, B_1_, C_1_, D_1,_ A_3_, B_3_, C_3_); 25 μm in D_4_ (applies A_2_, B_2_, C_2_, D_2,_ A_4_, B_4_, C_4_).

**Supplementary Figure 2**


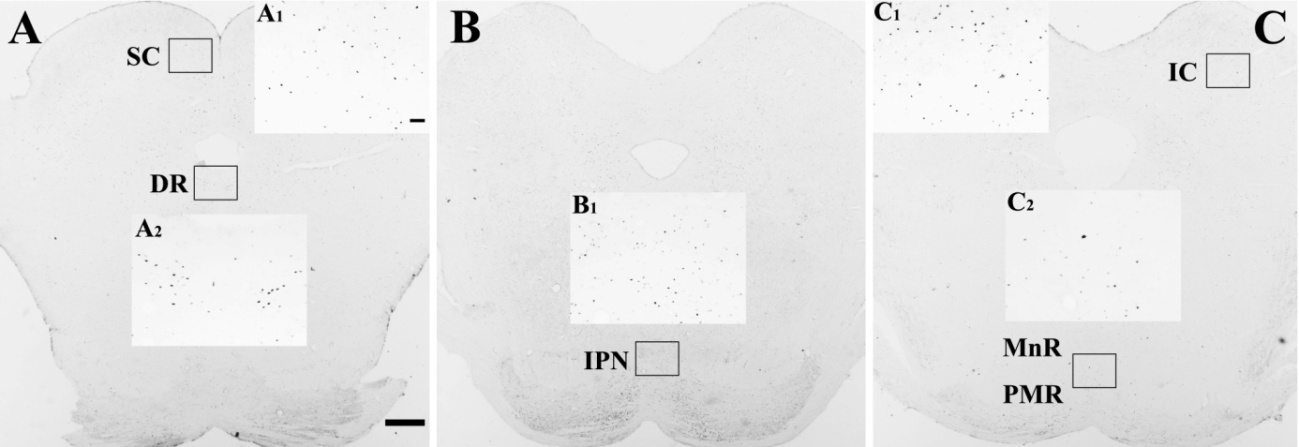
**Photomicrographs show the distributions of FOS-ir neurons in the midbrain after injecting 5% formalin into the hindpaw.** FOS-ir neurons can be seen in many areas of different PAG sections through DAB reaction (A, B, C). The framed areas SC and DR in (A), IPN in (B), IC and MnR, PMR in (C) are magnified in A_1_ and A_2_, B_1_, C_1_ and C_2_ respectively. Scale bars = 500 μm in A (applies B, C); 50 μm in A_1_ (applies A_2_, B_1_, C_1_, C_2_). DR, dorsal raphe nucleus; IC, inferior colliculus; IPN: interpeduncular nucleus; MnR: median raphe nucleus; PMR: paramedian raphe nucleus; SC: superior colliculus.
