# Supplementary figures and images for: Neurochemical properties of BDNF-containing neurons projecting to rostral ventromedial medulla in the ventrolateral periaqueductal gray
Source: Front Neural Circuits. 2014 Nov 20;8:137. doi: 10.3389/fncir.2014.00137 (PMC4238372; doi:10.3389/fncir.2014.00137)

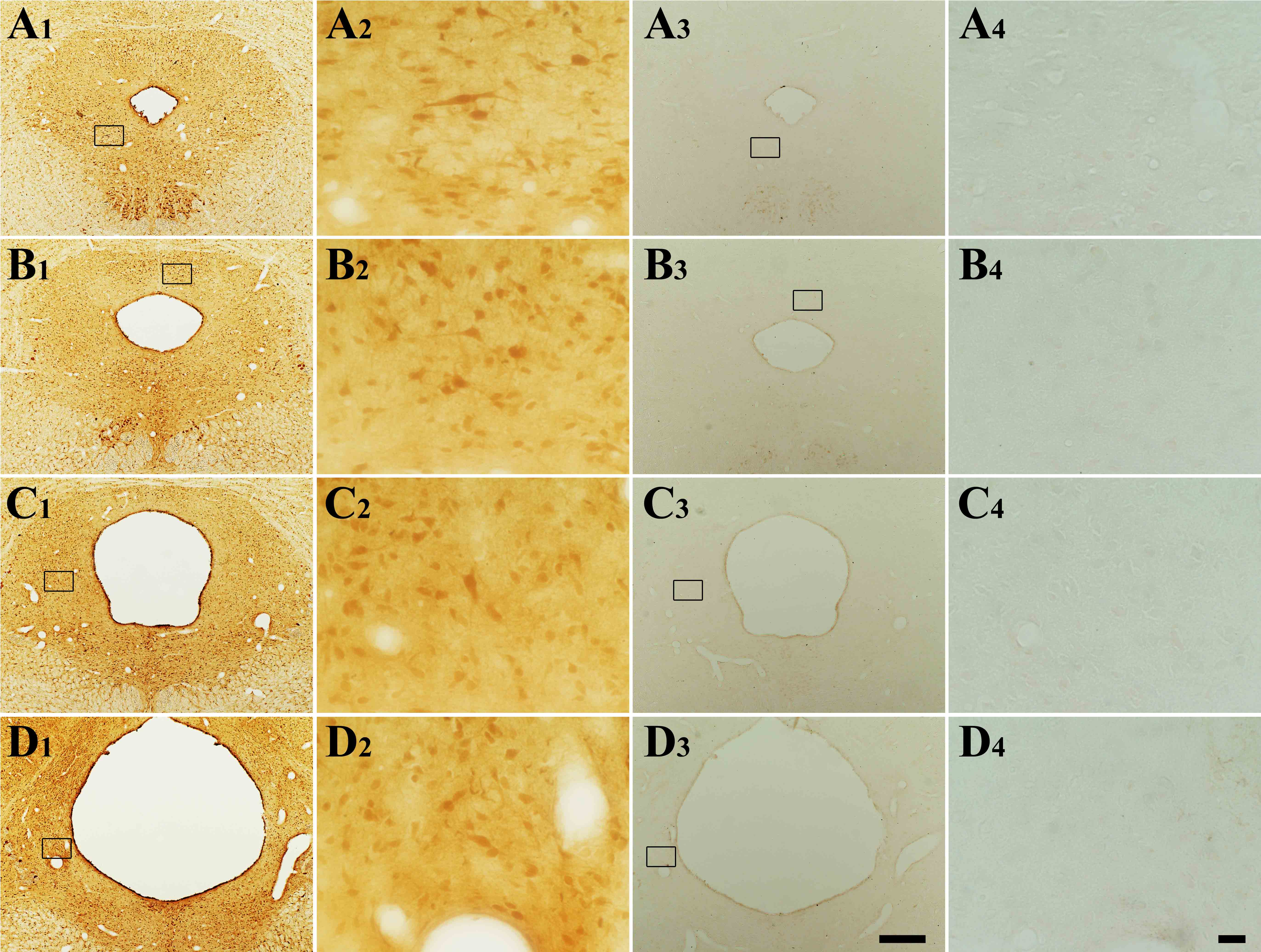

Supplement: Supplementary file 2 [file Image1.JPEG]

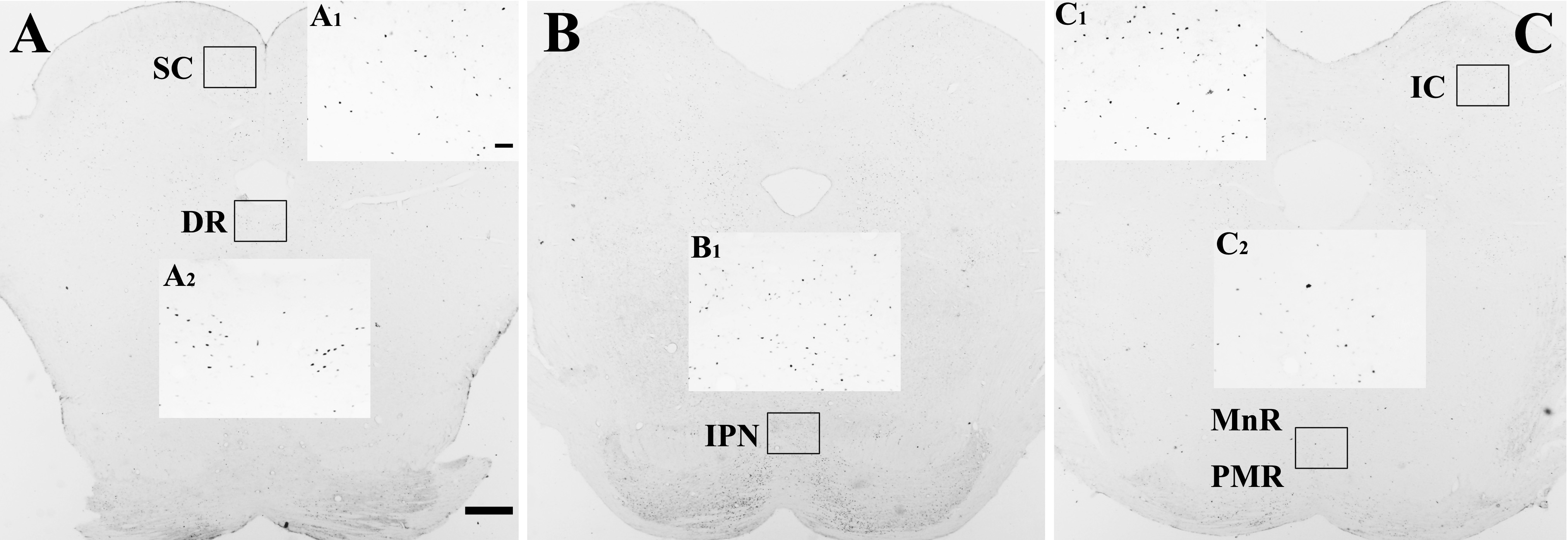

Supplement: Supplementary file 3 [file Image2.JPEG]
